# Case Report: Epstein–Barr Virus Encephalitis Complicated With Brain Stem Hemorrhage in an Immune-Competent Adult

**DOI:** 10.3389/fimmu.2021.618830

**Published:** 2021-02-25

**Authors:** Lingtong Huang, Xuan Zhang, Xueling Fang

**Affiliations:** ^1^ Department of Critical Care Units, The First Affiliated Hospital, Zhejiang University School of Medicine, Hangzhou, China; ^2^ Department of Infectious Disease, The First Affiliated Hospital, Zhejiang University School of Medicine, Hangzhou, China

**Keywords:** Epstein-Barr virus, encephalitis, brainstem hemorrhage, next-generation sequencing, vasculitis

## Abstract

Encephalitis caused by Epstein-Barr virus infection is uncommon, but most patients have a good outcome after symptomatic treatment. The infiltration of mononuclear cells in blood vessels and necrosis resulting from the immune response to Epstein-Barr virus infection in a very small number of patients seem to be the main cause of death. We describe a fatal case of Epstein-Barr virus encephalitis diagnosed by next-generation sequencing in an immune-competent adult but progressed to brainstem hemorrhage.

## Introduction

Epstein-Barr virus (EBV) encephalitis is a rare type of viral encephalitis that is associated with hematopoietic stem cell transplantation (1, 2), solid organ transplant (3), and HIV infection (4–7). It usually occurs in children (8, 9), but it has also been reported in a few immune-competent adults (10). EBV encephalitis typically has a good prognosis (6, 11–13), and very few patients develop serious neurological sequelae (14) or die (15). However, EBV encephalitis is difficult to diagnose because its clinical manifestations are complex and diverse. Most patients with EBV encephalitis are admitted to the hospital due to mental symptoms (15). EBV encephalitis usually occurs in the brain stem (9, 12, 16, 17), cerebellum (9), thalamus (18), basal ganglia (12), optic nerve (8, 12), and spinal cord (12). The most frequent clinical features of EBV encephalitis are mental deterioration (19), reversible parkinsonism (20, 21), vasculitis (4, 9, 15), and bleeding (3, 9).

In adults, there are no reports of brainstem hemorrhage caused by EBV encephalitis before. Here, we describe the first fatal case of EBV encephalitis diagnosed by next-generation sequencing (NGS) in an immune-competent adult and soon progressed to brainstem hemorrhage.

## Case Report

**Figure 1:**
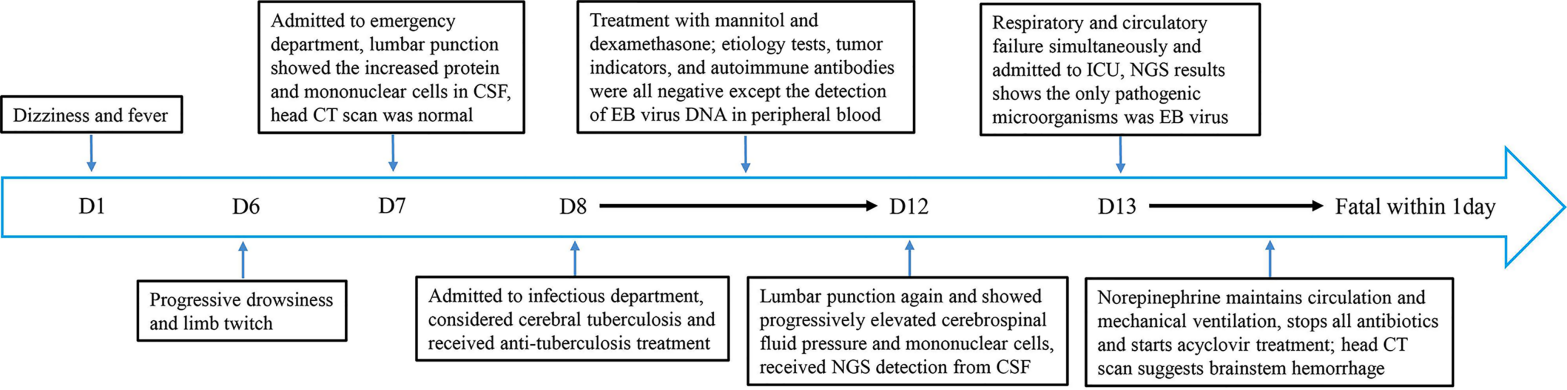
Timeline depicting the disease course of the patient. The timeline illustrates the different events in the course of the patient’s treatment and disease progression.

A 59-year-old man developed dizziness and fever (maximum body temperature, 39.0°C) 6 days ago without obvious causes and was not given any treatment. He developed several seizures 1 day ago and was brought to the emergency department (ED) of our hospital by his family, as shown in the timeline in **Figure 1**. The patient was in good health, had not traveled, and had no history of taking medication before, and he did not drink or smoke.

**Figure 2:**
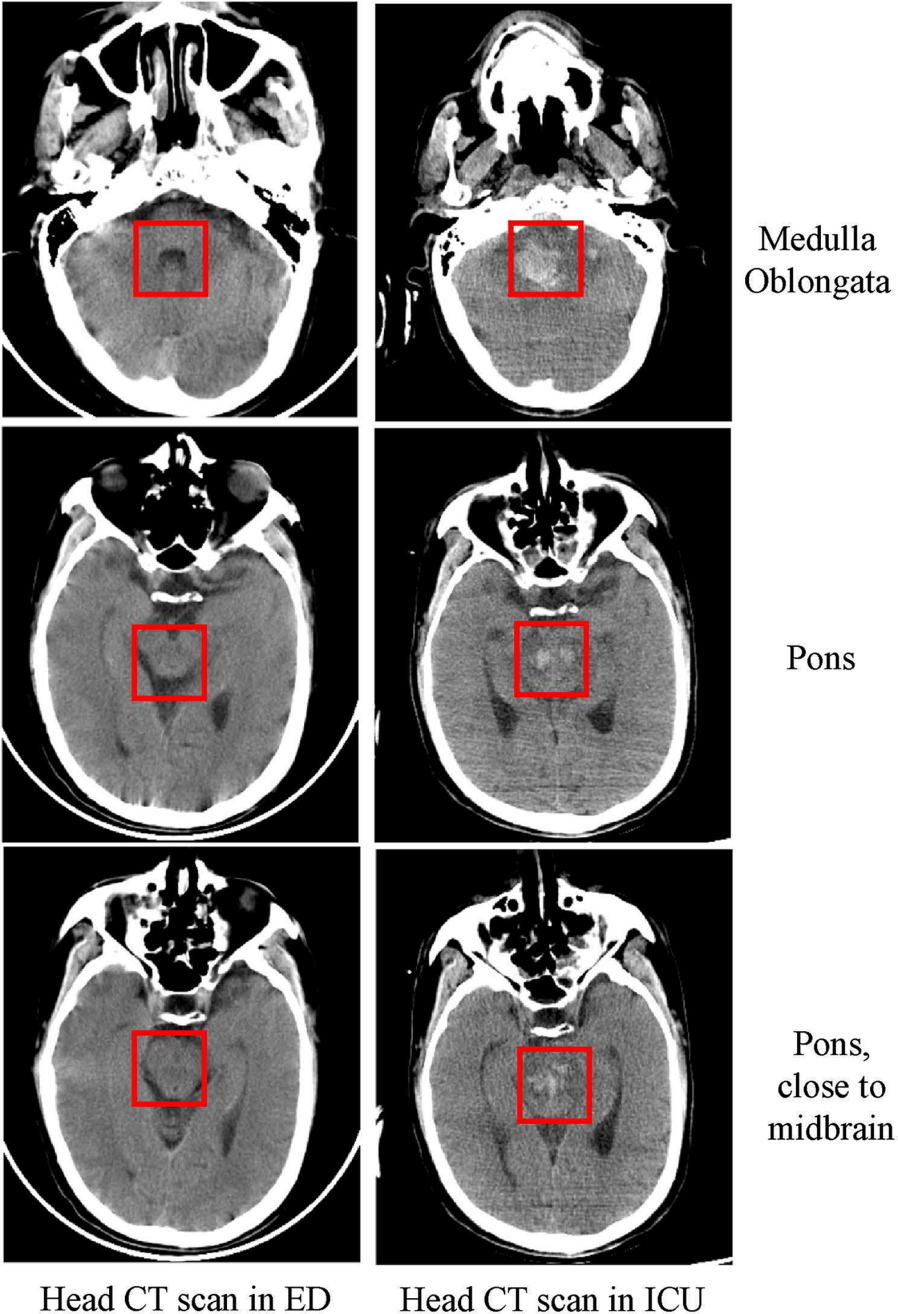
Head CT scan in the ED and ICU. Head CT scan showing that as the disease progressed within five days, the patient had extensive bleeding from the medulla oblongata to the pons as marked in the figure.

When he was brought to the ED, he was still unconscious and had a GCS score of E3V1M3 and meningeal irritation signs. An initial lab exam showed normal liver chemistry, kidney chemistry, and infection parameters (CRP 4.7 mg/L; PCT 0.13 ng/ml; ESR, 3 mm/h) (**Table 1**). There was no other evidence to prove the existence of infection, except for the fever. We performed a lumbar puncture under normal open pressure. The cerebrospinal fluid (CSF) pressure was 250 mm H_2_O, and CSF was colorless and not turbid. Analysis of CSF showed a red blood cell count of 12×10^6^/L and a mononuclear cell count of 28×10^6^/L (75% lymphocytes and 25% monocytes) (**Table 1**). CSF chemistry showed that the protein content increased to 1.982 g/L (normal value, 0.15–0.45 g/L), and the glucose and the Cl^-^ levels were normal. A head CT scan showed no abnormal signs (**Figure 2**), and an abdomen CT scan was not indicative of liver or spleen enlargement.

**Table 1:** Summary of inflammatory markers and CSF examination during the whole hospitalization course.

	Temp (°)	WBC (10^9/L)	CRP (mg/L)	PCT (ng/ml)	Examination of Cerebrospinal Fluid				
					Protein (g/L)	Glu (mmol/L)	Cl^-^(mmol/L)	Pressure (mmH_2_O)	Mononuclear cell (10^6/L)
HD1	36.4	10.8	4.7	0.13	1.982	2.4	112	250	28
HD2	38.4	9.1	10.3	0.2	1.087	3.4	117	250	130
HD3	37.1	13	/	/	/	/	/	/	/
HD4	38.6	11.2	/	/	1.010	4	114	310	200
HD5	38.8	12.7	58.3	0.26	/	/	/	/	/
HD6	37.8	12.5	54.9	0.24	/	/	/	/	/

HD, hospitalization day; Temp, Temperature; WBC, white blood cell; CRP, C-reactive protein; PCT, procalcitonin.

The patient was transferred from the ED to the infection department, and cerebral tuberculosis was suspected. Over the next three days, he was administered anti-tuberculosis treatment, moxifloxacin for anti-infection treatment, and mannitol three times a day to reduce intracranial pressure. Additionally, autoimmune encephalitis was suspected, and the patient was intravenously administered 10 mg dexamethasone. A series of blood tests conducted over the next three days revealed negative results for the T-spot test, cytomegalovirus DNA, bacteria assay (blood culture and cerebrospinal fluid culture), fungal assay (*Cryptococcus neoformans* antigen assay), virus assay in the blood (hepatitis A, hepatitis B, hepatitis C, hepatitis D, Cytomegalovirus, and herpes simplex virus), autoantibodies (antinuclear antibody, double-stranded DNA, soluble nuclear protein antibody, RNP, Sm, SSa, ssa52, anti-SSB, anti-Scl-70, anti-PM-scl, anti-JO-1, centromere antibody, anti-PCNA, small nuclear body antibody, Rib, APS-related antibody, anti-mitochondrial antibody, IgG4, MPO, ANCA, and anti-*N*-methyl-d-aspartate antibody), and tumor markers (AFP, CEA, CA125, CA199, ferritin, and prostate-specific antigen). The EBV DNA detected with primers targeting BamHI-W was 1,300 copies/ml in blood, anti-EBV capsid antigen IgM was negative, and anti-EBV capsid antigen IgG was positive blood. The patient’s CRP levels were normal, and PCT levels were below 0.5 ng/ml.

On the fourth day of hospitalization, the patient was still unconscious. Lumbar puncture was performed again under normal opening pressure. Additionally, pathogen detection in the CSF was performed by NGS. The NGS detection method was the same as we described before (22). The measured CSF pressure was 310 mm H_2_O, and the CSF was still colorless and not turbid. Analysis of CSF revealed a red blood cell count of 30×10^6^/L, an increase in the mononuclear cell count to 200×10^6^/L (70% of lymphocytes), a decrease of protein content to 1.010 g/L, and normal glucose and Cl^-^ levels.

**Figure 3:**
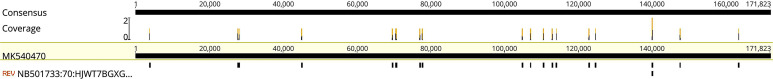
Mapping results of EBV reads in cerebrospinal fluid (CSF). Mapping results of EBVs in the CSF to EBV reference genome MK540470.

On the fifth day of hospitalization, circulatory failure and a decrease in oxygen saturation were observed, and the patient was transferred to the intensive care unit (ICU) with a GCS score of E1V1M3. After transfer to the ICU, tracheal intubation was performed. The NGS results showed that the CSF was positive for the EBV (**Figure 3**) and negative for fungi, bacteria, and parasites. These findings were indicative of EBV encephalitis. Therefore, all antibiotic treatments were discontinued, and 0.5 g acyclovir was intravenously administered three times a day.

After the patient was stable, a head CT examination was performed that revealed a massive hemorrhage in the brainstem that could not be surgically removed (**Figure 2**). The patient’s blood pressure dropped again within 1 day of admission to the ICU, and this was accompanied by a decrease in oxygen saturation and unequal pupil size. Soon after, the patient succumbed to the fatalities.

## Discussion

In this study, we describe a rare case of an immune-competent patient with EBV encephalitis in whom the intracranial pressure continued to increase progressively despite mannitol treatment. The case was complicated by brainstem hemorrhage in a short time, and the patient eventually died. In this case, cancer, other pathogen infections, autoimmune encephalitis, and other conditions were ruled out, and NGS showed that the CSF was positive for EBV DNA. Therefore, EBV encephalitis was diagnosed.

As described in previously reported cases of EBV encephalitis (6, 17–19), CSF pressure, mononuclear cell count, and protein content in the CSF increased after infection onset, but the glucose and Cl^-^ levels were normal which was also observed in this case. EBV encephalitis has no typical symptoms, and the results of CSF examination are similar to those for cerebral tuberculosis (4, 12). The presence of EBV DNA in the blood is very common in hospitalized patients (clinically relevant cutoff value = 2,000 copies/ml) (23), so we did not consider EBV encephalitis for the viral load of 1,300 copies/ml at the beginning. Therefore, in this case, it is inevitable that the patient was initially misdiagnosed before the NGS results were obtained. Some patients also test positive for autoantibodies such as the anti-*N*-methyl-d-aspartate antibody (24), and as a result, anti-N-methyl-D-aspartate receptor encephalitis was diagnosed (25, 26). In this case, however, the results of anti-*N*-methyl-d-aspartate antibody were negative.

In most cases, EBV encephalitis is accompanied by liver and spleen enlargement (15, 27). However, some patients may not have an enlarged liver and spleen (19), which provides a challenge for diagnosing EBV encephalitis. In the early stage of the disease, CT does not show abnormalities, and MRI examinations often show increased signal intensity on T2-weighted imaging sequences on both temporal lobes (12, 15, 19, 28). In the present case, the CT images did not show any abnormalities, except for the CT scan taken later that showed cerebral hemorrhage. For suspected intracranial infection or autoimmune encephalitis, an MRI examination is necessary. MRI examination can indicate viral encephalitis, but imaging examination cannot pinpoint the exact pathogen for us. Due to the patient’s consciousness disturbance when admitted to ED, it is impossible to inquire whether the patient has metal implants, and the MRI examination is extremely risky. Besides, the patient has been given monitoring measures, including ECG monitors in ED and infection department, so we could not perform MRI.

The autopsy results of patients who died of EBV encephalitis show mononuclear cell infiltrates in the perivascular and necrotic hemorrhagic focus in the brain (9, 15, 29). Therefore, the pathogenesis of EBV encephalitis is more inclined towards inflammation caused by the immune response rather than the virus itself (30), and most patients with EBV encephalitis are treated with intravenous immunoglobulins (31) and steroids (15, 21). Currently, ganciclovir (6, 13, 27) and acyclovir (21, 27) are often used for treatment, but there is no standard treatment strategy for EBV encephalitis. Although we used steroids before diagnosing EBV encephalitis, we were still unable to prevent the disease’s rapid progression. EBV encephalitis may be a self-limiting disease (32), and no double-blind, randomized controlled clinical trial has proven the effectiveness of these drugs. The only treatment that can eliminate EBV infection is hematopoietic stem cell transplantation (33), but this can be very difficult to practice.

In the present case, the patient was admitted to the hospital because of neurological symptoms, but a head CT scan taken at the admission time showed no abnormalities. The patient’s condition continued to deteriorate during hospitalization, and brainstem hemorrhage occurred on the fifth day. The patient’s condition was similar to that described in a child: extensive bleeding points appeared in the brain stem after EBV encephalitis (9). In this case, we speculate that the brainstem hemorrhage may be related to EBV-induced vasculitis, which ultimately led to simultaneous failure of the patient’s breathing and circulation. The limitation of this case is that we cannot perform an autopsy to confirm it, and we can only speculate by CT findings and the increasing number of mononuclear cells in CSF.

Although the patient died quickly in the end, we believe that NGS testing may become an effective means to rule out non-viral infections such as fungi, bacteria, tuberculosis, and some parasites. At the same time, after rule out the possibility of tumors, steroids and intravenous immunoglobulins seem to be an effective salvage treatment in some critical care patients. There are many challenges for NGS, including data analysis, expensive cost, and time required. In this case, NGS’s turn-around time was 24 h, and the cost was approximately 300 dollars. Actually, in our hospital, EBV qPCR is only tested once a day in the morning, so qPCR’s turn-around time still needs 24 h. Besides, except EBV, we have to determine the presence of Herpes simplex virus, Cytomegalovirus, and other viral DNA if we use qPCR to identify the pathogenic microorganisms in CSF, and the cost was approximately 200 dollars, which is similar to the NGS test. Therefore, in our view, NGS is an acceptable test for critically ill patients with unknown pathogens. It should be noted that if blood vessels are damaged during lumbar puncture, the harvested cerebrospinal fluid samples may be contaminated with EBV DNA from the blood. During the NGS test, the specimen will be centrifuged to reduce the impact of its own DNA, so we can only retrospectively get the results of free DNA in the patient’s cerebrospinal fluid. Single-cell sequencing of cerebrospinal fluid to determine what changes have occurred in the immune cells of CSF may help us clarify the pathogenesis of EBV encephalitis and possible therapeutic targets. Finally, the patient’s family refused the autopsy, and we could not get the pathological results to get the most direct evidence of EBV infection in the brain, which was the limitation of this case.

In conclusion, this is the first reported case of EBV encephalitis complicated by brainstem hemorrhage in an immune-competent adult who eventually succumbed to the fatalities. Based on the findings, it is recommended that in patients with signs of encephalitis, NGS of CSF be used to quickly rule out the diagnosis of encephalitis caused by other pathogenic microorganisms and confirm the diagnosis of EBV encephalitis. Simultaneously, start intravenous immunoglobulins and steroid therapy as soon as possible may be beneficial to patients with life-threatening EBV encephalitis.

## Data Availability Statement

The original contributions presented in the study are included in the article/supplementary material. Further inquiries can be directed to the corresponding author.

## Ethics Statement

The studies involving human participants were reviewed and approved by Ethics Committee of the First Affiliated Hospital of Zhejiang University. The patients/participants provided their written informed consent to participate in this study.

## Author Contributions

LH and XZ wrote the case report, drafted the manuscript, and prepared the figures. XF critically reviewed the final manuscript. All authors contributed to the article and approved the submitted version.

## Funding

The work was supported by the National Science & Technology Major Project of China (grant #2017ZX10204401 to XZ) and Zhejiang Provincial Natural Science Foundation of China (grant #LQ19H190001 to XZ). The funders had no role in the decision to publish or prepare the manuscript. We thank Dr. Xia Jin from IngeniGen XunMinKang Biotechnology Inc. for her work on NGS data processing and analysis in this manuscript.

## Conflict of Interest

The authors declare that the research was conducted in the absence of any commercial or financial relationships that could be construed as a potential conflict of interest.
